# Live Imaging of Cysteine-Cathepsin Activity Reveals Dynamics of Focal Inflammation, Angiogenesis, and Polyp Growth

**DOI:** 10.1371/journal.pone.0002916

**Published:** 2008-08-13

**Authors:** Elias Gounaris, Ching H. Tung, Clifford Restaino, René Maehr, Rainer Kohler, Johanna A. Joyce, Hidde L. Plough, Terrence A. Barrett, Ralph Weissleder, Khashayarsha Khazaie

**Affiliations:** 1 Center for Molecular Imaging Research, Massachusetts General Hospital and Harvard Medical School, Charlestown, Massachusetts, United States of America; 2 Division of Gastroenterology, Northwestern University Feinberg School of Medicine, Robert Lurie Comprehensive Cancer Center, Chicago, Illinois, United States of America; 3 Cancer Biology and Genetics Program, Memorial Sloan Kettering Cancer Center, New York, New York, United States of America; 4 Whitehead Institute, Massachusetts Institute of Technology, Cambridge, Massachusetts, United States of America; 5 The Methodist Hospital Research Institute, Weill Cornell Medical College, Houston, Texas, United States of America; 6 Department of Stem Cell and Regenerative Biology, Harvard Stem Cell Institute, Harvard University, Cambridge, Massachusetts, United States of America; 7 Howard Hughes Medical Institute, Cambridge, Massachusetts, United States of America; University of California Merced, United States of America

## Abstract

It has been estimated that up to 30% of detectable polyps in patients regress spontaneously. One major challenge in the evaluation of effective therapy of cancer is the readout for tumor regression and favorable biological response to therapy. Inducible near infra-red (NIR) fluorescent probes were utilized to visualize intestinal polyps of mice hemizygous for a novel truncation of the Adenomatous Polyposis coli (APC) gene. Laser Scanning Confocal Microscopy in live mice allowed visualization of cathepsin activity in richly vascularized benign dysplastic lesions. Using biotinylated suicide inhibitors we quantified increased activities of the Cathepsin B & Z in the polyps. More than ¾ of the probe signal was localized in CD11b^+^Gr1^+^ myeloid derived suppressor cells (MDSC) and CD11b^+^F4/80^+^ macrophages infiltrating the lesions. Polyposis was attenuated through genetic ablation of cathepsin B, and suppressed by neutralization of TNFα in mice. In both cases, diminished probe signal was accounted for by loss of MDSC. Thus, *in vivo* NIR imaging of focal cathepsin activity reveals inflammatory reactions etiologically linked with cancer progression and is a suitable approach for monitoring response to therapy.

## Introduction

Colonic and intestinal adenocarcinomas arise as a direct result of the loss of function of the adenomatous polyposis coli (APC) gene and stabilization of β-catenin. Rodent models of hereditary colon cancer faithfully reproduce the histopathology of familial adenomatous polyposis coli (FAP) [1], [2] and provide opportunities for investigating secondary events that modulate genetic predisposition to colon cancer [3]. Accumulating evidence suggests that inflammation has causative roles in carcinogenesis [4]. While chronic inflammation can predispose to DNA damage and carcinogenesis, there is evidence to suggest that inflammation is a necessary component of tumor growth. In line with this notion, treatment of APC defective Min mice [5] with cyclooxigenase-2 (COX2) inhibitors results in a transient suppression of polyposis [6], [7], an observation that parallels the response of colon cancer patients to similar treatments [8]. Furthermore, anti-TNFα, or the transfer of CD4^+^CD25^+^CD45RB^low^ regulatory T (Treg) cells, both hinder polyp growth in mice [9]. Together these observations strongly argue in favor of a causative link between inflammatory reactions and genetically induced colon cancer, opening possibilities for monitoring and targeting cancer associated inflammation for diagnostic and therapeutic purposes.

Proteolytic enzymes play essential roles in tumor growth, angiogenesis, and invasion. Cathepsins of the cysteine protease family and in particular cathepsin-B are commonly active in the tumor microenvironment, contributing to the regulation of angiogenesis and invasion during cancer progression [10], [11]. We have shown that optical *in vivo* imaging of cathepsin B activity using near infra-red mechanism-based probe allows for highly sensitive detection of adenomatous polyps in mice with direct reflectance imaging [12], [13]. The cathepsin inducible fluorescent probe (ProSense 680) is a composite polymer containing a poly-L-lysine backbone; on which quenched NIR (excitation 675 nm, emission 694 nm) fluorophore and several polyethyleno-glycol side-chains are attached. ProSense 680 is preferentially hydrolyzed by cathepsin B, but it can be activated through proteolysis by other cathepsins and other related proteases [14]. Using this approach, mouse polyps were specifically revealed by reflectance imaging [13], [14], [15] and by *in vivo* fluorescence endoscopy [16]. However, the cellular source of signal and biological meaning of the protease activity has remained enigmatic.

Here we used targeted *in vivo* analysis of cysteine cathepsins with ProSense 680. To investigate how specifically the probe activity demarcates areas of dysplasia and the relevance of probe signal to biological activity within the tumor we used the prototype Olympus IV 100 scanning LASER intravital microscope to image intestinal lesions in a new mouse model of hereditary polyposis, APC^Δ468^ mice. We report that probe activation reflects the local density of pro-inflammatory cells infiltrating the lesion and amount of associated active enzyme at the tumor site. Furthermore, using cathepsin B deficient APC^Δ468^ (Ctsb^−/−^ APC^Δ468^) mice, as well as, anti-inflammatory treatments, we provide evidence for a causative link between protease activity, inflammation and polyp growth. Altogether, the present study shows that NIR imaging of pre-neoplastic lesions using near infra red mechanism-based probes is a viable approach to detect biological activities etiologically connected with progressive tumor growth, and provides opportunities for monitoring biological response to effective therapy.

## Results

### Mouse APC^Δ468^ Model: Morphology and Histopathology of Adenomatous Polyps

A novel model of hereditary polyposis was generated by targeted deletion of exons 11 and 12 of the adenomatous polyposis coli (APC), causing truncation of the gene product at codon 468. The resulting mice (APC^Δ468^) were backcrossed to C57BL/6J for at least 12 generations. Adenomatous polyps were found in abundance in the small intestine of the hemizygous APC^Δ468^ mice as early as 5 weeks of age, most frequently in the distal ileum (Fig. S1a&b). Colonic polyps were less frequent and increased with age. Morphologically, the polyps were undifferentiated Fig. S2a&b), were polyclonal (Fig. S2c), tubovillar (Fig. S2d&e), and were composed of tall hyperchromatic disorderly cells with cigar-shaped nuclei (Fig. S2f).

**Figure 1:**
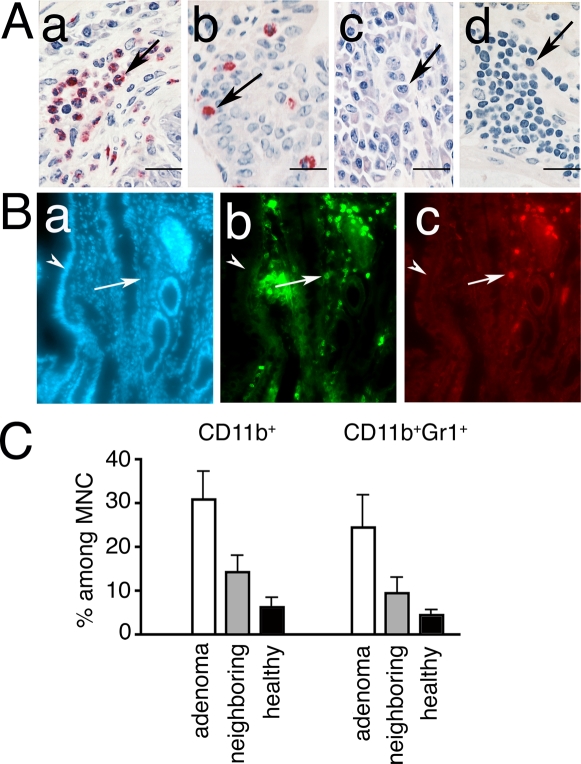
Infiltration of polyps by pro-inflammatory cells. (Α) Histology of polyps from APC^Δ468^ mouse. (a–b) CEA stained paraffin sections counterstained with Gill's II Hematoxylin; (c&d) methylene blue staining; arrows point to (a) granulocytes, (b) mast cells, (c) plasma cells, (d) lymphocytes. (B) Immuno-fluorescence of polyps from APC^Δ468^ mice. Cryosections were stained with (a) DAPI, (b) CD11b-AlexaFluor 488, and (c) Gr1-AlexaFluor 594. Arrows point to polyp, and arrowheads to the adjacent healthy villus; note accumulation of CD11b^+^ cells and/or Gr1^+^ cells in the polyps. (C) FACS analysis of leukocytes prepared from micro-dissected polyps, and from adjacent tissue (n = 4), and of intestinal tissue from age-matched healthy control mice (n = 3). Mean values and SEM are shown for frequencies of CD11b^+^ and of CD11b^+^Gr1^+^ cells in 6 month-old mice.

**Figure 2:**
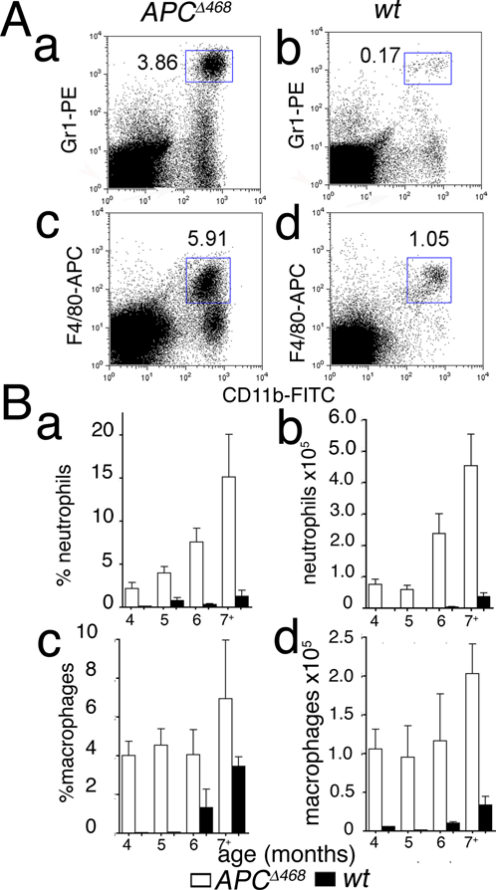
Flow cytometric analysis of polyp infitrate. (A) FACS analyses of pro-inflammatory cells. (a) Exemplar FACS analyses of mononuclear cells prepared from the intestine of (a&c) a 5 month-old APC^Δ468^ mouse and (b&d) an age matched *wt* C57BL/6J mouse; note increases in the frequencies of (a) CD11b^+^Gr1^+^ and (c) CD11b^+^F4/80^+^ myeloid type cells in the polyposis intestine, compared to *wt* tissue (b&d respectively). (B) Summary of FACS analysis of MNCs from *wt* (black bars) or APC^Δ468^ (open bars) intestine, showing mean frequencies and absolute numbers of cells with SEM values; n = 6 for APC^Δ468^, n = 3 for *wt* control mice, per age group.

Closer histological examination revealed a rich infiltrate of hematopoietic cells, including granulocytes, mast cells, plasma cells, and lymphocytes (Fig. 1a–d). Two distinct populations of myeloid cells were significantly increased in the lesions: CD11b^+^GR1^+^ (MDSC), and CD11b^+^F480^+^ macrophages (Fig. 1B&C). The MDSC infiltrate increased with age and tumor load (Fig. 2Aa compare with 2Ab; 2Ba&b), while macrophage density was elevated abruptly and remained high (Fig. 2Ac compare with 2Ad; Bc&d). In contrast, the intestine of ageing *wt* mice showed little infiltration by MDSC, and had delayed increase in macrophages (Fig. 2B, black bars). As the APC^Δ468^ mice aged, anemia and systemic inflammation developed contributing to a notable increase in the size of the spleen (Fig. S3a), which was in part accounted for by infiltrating MDSC and macrophages (Fig. S3b&c).

### Near infra red mechanism-based probes accurately mark areas of dysplasia

Aged mice showing early signs of cahexia were injected with the ProSense 680 (2 nmoles/mouse) 24 hours before the imaging session. To image angiogenesis we employed the constitutively emitting AngioSense 750 probe and injected the mice just before the imaging session.

**Figure 3:**
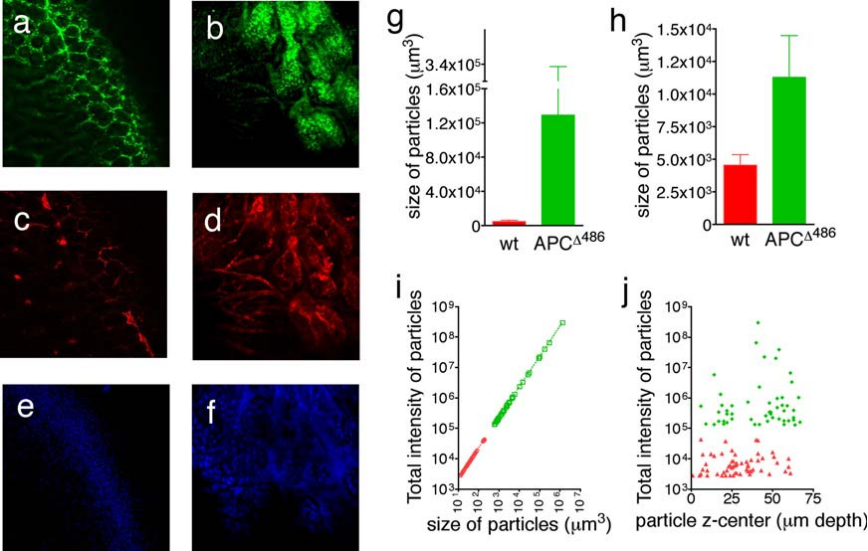
*In vivo* molecular imaging of polyps in APC^Δ468^ mice. A small loop of the intestine of each APC^Δ468^ and *wt* animals was surgically exposed and underwent laser scanning Intravital Fluorescent Microscopy (IVFM) 24 hour after injection with the ProSense-680. ProSense-680 image of cathepsin activity in (a) healthy intestine, (b) APC^Δ468^ polyp. AngioSense 750 image of vasculature in (c) healthy intestine, (d) APC^Δ468^ polyp. Spectrally resolved auto-fluorescence of (e) healthy intestine, (f) APC^Δ468^ polyp at 505–510 nm. Z-stack slice 42 for the healthy intestine and slice 51 for the APC^Δ468^ polyp were imaged using an UplanApo 10× objective with 2× electronic zoom (pixel size 1.05 µm with resolution 2.42 µm). (g) The mean volume of particles visualized at 694 nm (ProSense-680); green bar represents APC^Δ468^ polyps (128,900±104,800 µm^3^ (n = 92)), red bar healthy intestine (4,538±797.5 µm^3^ (n = 81)). (h) The mean volume of particles visualized at 790 nm (AngioSense-750); green bar represents vasculature in polyps (11270±3207 µm^3^, n = 101), red bar represents vasculature in healthy intestine (4538±798 µm^3^, n = 81). Calculated with the Image J “3D particle analysis” plug-in. (i) Linear regression, correlating the size of ProSense-680 particles in polyps (green dots, 1/slope = 0.004540, r^2^ = 1) with those in healthy intestine (red dots, 1/slope = 0.004539, r^2^ = 0.9999); note that the increase in intensity corresponds to the increase in the number of cathepsin active cells. (j) “Calculated centers of intensity” of particles in the z-axis of the APC^Δ468^ adenoma (green diamonds) and healthy intestine (red triangles); note distribution throughout the z- stack, and that the total intensity of the particles in the APC^Δ468^ adenoma is at least 2 orders of magnitude higher (mean total intensity 1.322×10^7^±8.881×10^6^ units) than the particles in the *wt* intestine (mean total intensity 8592±1257 units, *P*<0.0001 one sample t test).

Mice were anesthetized, a short loop of the small bowel was surgically exposed and individual polyps together with surrounding tissue as well as healthy bowel tissue were imaged with the prototype Olympus IV 100 scanning LASER intravital microscope. Images were collected using dry ×4 and ×10 lenses in z-stacks, from the tips of the luminal border towards the submucosa. After spectral separation, three channels, namely 694 nm, 790 nm, and 505–510 nm, were used to collect images from the cathepsin inducible ProSense 680 (Fig. 3a&b), the vascularity-contrasting agent, AngioSense 750 nm (Fig. 3c&d), and autofluorescense (Figure 3e&f) respectively. Images were recorded from 1 µm thickness sections, down to more than 100 sections depth. The collected composite files of the z-stacks were analyzed with the Image J software.

In healthy bowel, the ProSense 680 image revealed a canonical honeycomb-like distribution of the signal depicting the axial image of the intestinal villi (Fig. 3a). The AngioSense 750 (Fig. 3c) and auto-fluorescence at 505–510 nm revealed similar honeycomb distributions, tracing the blood vessels and stroma (Fig. 3e). The image of micro-adenomas was drastically different, with broad areas of high signal intensity demarcating areas of dysplasia (Fig. 3b), and rich arrays of micro-vessels supplying the lesions. Inside the tumor, the vessels expanded into large dead-end structures typical of mother vessels that are often characteristic of tumor vasculature (Fig. 3d) [17], [18]. Tumor stroma was strongly autofluorescent (Fig. 3f, Fig. S4).

The increased signal of the ProSense 680 in microadenoma is attributed to increased local cathepsin activity. To demonstrate this, we imaged microdenoma 24 hours after injecting mice with 2 nmoles ProSense 680 and 2 nmoles ProSense-control 750. The Prosense-control 750 cannot be hydrolyzed by cathepsins and the fluorophore remains quenched.since its backbone is made with poly-D-lysine. Accordingly, while the ProSense 680 signal was readily visible within microadenoma (Fig S5a) the ProSense control 750 failed to become activated (Fig. S5b; see merged Fig. S5a). In a typical experiment, the ratio of the mean intensities of the ProSense 680 to ProSense control 750 throughout the z-stack within a polyp was 49±1.4 (168 slices, 1 µm/slice), while in the adjacent healthy tissue this ratio was 2.1±0.01 (*p*<0.0001 one tailed t test with Welch correction, n = 168 slices) (Fig. S5d). These observations strongly suggest the specific activation of Prosense-680 through proteolytic cleavage.

To investigate whether the increased signal was due to more activity per cell or a greater number of cells with the same individual activity, we analyzed the volume and total intensity of the ProSense 680^+^ particles. Their mean volume in the polyp z-stack (128900±104800 µm^3^, n = 92) was over 28 times that in the healthy intestine (4538±797.5 µm^3^, n = 81, Fig. 3g). The increase in total intensity of the ProSense-680^+^ particles was directly proportional to the increase in volume of signal (Fig. 3i; 1/slope = 0.004540 for the APC^Δ468^ polyps r^2^ = 1, total number of values 51, and 1/slope = 0.004539 for the healthy intestine r^2^ = 0.9999, total number of values 60), suggesting that the increase in signal intensity was due to an increase in the numbers of cathepsin active cells. In agreement with this conclusion, we found no significant difference in the “specific intensity” (intensity per µm^3^) of the particles found in APC^Δ468^ mice (242.9±0.01 units/µm^3^), and in *wt* mice (242.2±0.413 units/µm^3)^, the *P* value between the sets being 0.1233 in the unpaired t test with Welch's correction (the values of the size of the particles and their intensity were collected from the original 16-bit image). Furthermore, it was obvious that the centers of the ProSense-680^+^ particles were distributed throughout the acquired slices of z-stacks, and were uniformly larger in volume and therefore brighter by about 2 orders of magnitude in the polyps (1.321*10^7^±8.873*10^6^) as compared to the healthy surrounding tissue (8592±1257) (Fig. 3j; Suppl Video S1). Similar analysis of AngioSense-750 showed that the mean volume of the vessels in polyps was nearly 2.5 times higher (11270±3207 µm^3^, n = 101) than the mean volume of the vessels in the normal intestine tissue (4538±798 µm^3^, n = 81, Fig. 3h). Thus, increased vessel volume provides an independent means of visualizing early dysplasia, in accordance with published literature that links cathepsin activity with neovascularization [^11^], [^19^], [20].

### ProSense 680 is activated by MDSC and macrophages

**Figure 4:**
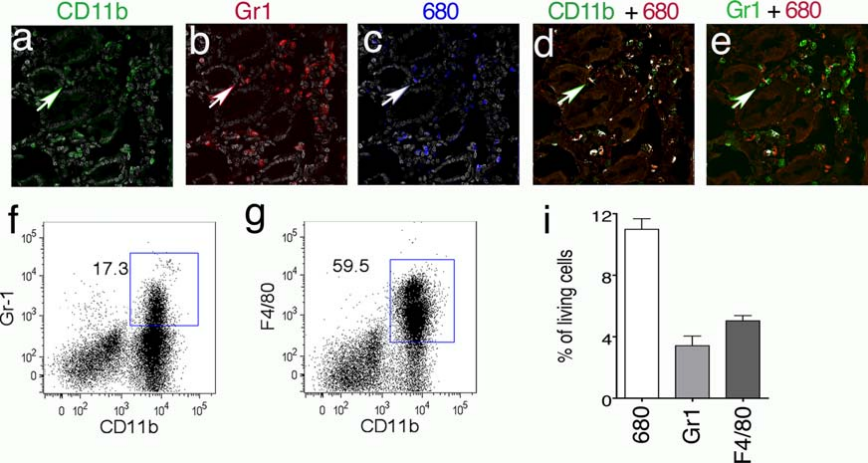
The cellular source of cathepsin activity. Cryosections of ProSense-680 *in vivo* stained intestine from APC^Δ468^mice were stained with antibodies to CD11b (AlexaFluor 488), Gr1 (AlexaFluor 594) and DAPI. The merged images of CD11b with DAPI (a, CD11b green, DAPI gray), Gr1 with DAPI (b, Gr1 red, DAPI gray), and ProSense-680 with DAPI (c, ProSense-680 blue, DAPI gray) were produced with the “RGB gray” plug-in of Image J. The “colocalization finder” plug-in produced images where the colocalized pixels appear white while the ProSense-680 was red (d&e), the CD11b was green (d, colocalization analysis of ProSense-680 and CD11b staining) and the Gr1 was green (e, colocalization analysis of ProSense-680 and Gr1 staining). ×400 magnification. Arrows mark a CD11b^+^Gr1 ProSense 680^+^ cell. Representative FACS dot-plots of MNCs prepared from polyposis intestine and *ex vivo* stained with ProSense-680 followed by CD11b and Gr1 staining. The live MNCs were gated for ProSence-680^+^ cells, which were analyzed for CD11b^+^Gr1^+^ (f, MDSCs) and CD11b^+^ F4/80^+^ (g, macrophages) cells. Cumulative results of 6 FACS experiments showing % of CD11b^+^Gr1^+^ ProSense-680^+^ and CD11b^+^F4/80^+^ProSense 680^+^ among total infiltrating MNCs. Note that among the ProSense-680^+^ cells (11±0.69% of total MNCs) over 75% were either CD11b^+^Gr1^+^ (3.4±0.6% of total MNCs) or CD11b^+^F4/80 (5.0±0.34% of total MNCs).

To reveal the cellular source of cathepsin activity, APC^Δ468^ mice were stained *in vivo* by injecting i.v ProSense-680 24 hours prior to being sacrificed. The entire gut was then excised, washed, fixed and embedded in OCT and frozen for histology and immunofluorescence analyses. Immunofluorescence staining revealed overlap of both CD11b (AlexaFluor 488) and Gr1 (AlexaFluor 594) with ProSense-680 signal (Fig. 4a–e), in cells that were dispersed through out the polyp stroma. The Image J “colocalization finder” in conjunction with the “nucleus counter” plug-in was utilized to analyze the fluorescent images. Figure 4d shows the result of the co-localization between the ProSense-680^+^ (red) and CD11b^+^ (green) fluorescent images. Co-localized pixels were revealed as white spots, and correspond to the 22/42 CD11b^+^ cells and 22/36 ProSense 680^+^ cells. Figure 4e is the outcome of the colocalization analysis between ProSense-680^+^ (red) and the Gr1^+^ (green) fluorescent images. Again, co-localized pixels were revealed as white spots, and this time correspond to the 11/45 Gr1^+^ cells and 11/36 ProSense-680^+^ cells.

To further characterize cathepsin probe-active cells, total Mono-Nuclear Cells (MNCs) were prepared from intestine of 5-month-old APC^Δ468^ mice. These were incubated *ex vivo* with 0.2 nmoles/ml ProSense-680 for one hour and after cell surface staining subjected to FACS analysis. MDSC and macrophages accounted for over 75% of the ProSense^+^ MNCs (Fig. 4f&g). Altogether, 11±0.69%, of the living cells (mice n = 6) were stained with the cathepsin activated probe, of which 3.4±0.6 were MDSC and 5.0±0.34% macrophages (Fig. 4i). For control, the MNCs were incubated with 50 µg/ml JPM-565 that is a general cathepsin inhibitor in RPMI 1640 for an hour at 37°C in the presence of 5% CO_2_ and then stained with 0.2 nmoles/ml ProSense 680. This resulted in a marked reduction in the intensity of staining of CD11b^+^ cells by Prosense 680 (Fig. S6a). In a typical experiment the frequency of ProSense 680+ cells was reduced from 3.49% to 1.59% (Fig. S6b). This result indicates that cathpesin activity was responsible for the *ex vivo* staining of myleloid cells by Prosense 680.

### Cathepsin activity reports focal inflammatory reactions in dysplasia

**Figure 5:**
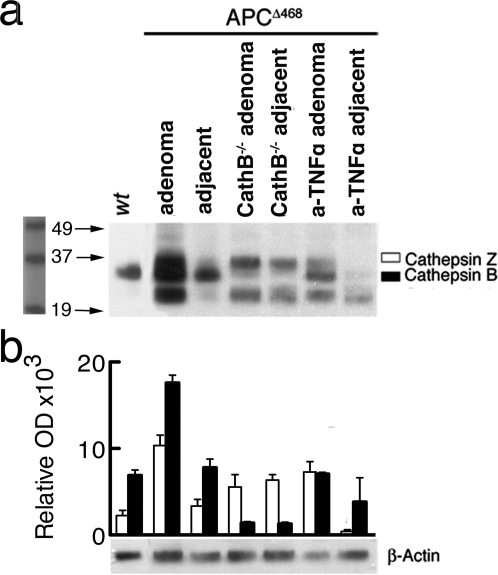
Quantification of active cysteine cathepsins using a specific active site directed probe. Polyps from APC^Δ468^, Ctsb^−/−^ APC^Δ468^, and anti-TNFα treated APC^Δ468^ mice were micro-dissected, pooled, and extracts were incubated with DCG-04 prior to electrophoresis on a 4–12% gradient SDS gel and western blotting; healthy adjacent regions were similarly analyzed. Active Cathepsins were visualized with the use of chemiluminescence reagents. (a) A representative blot. (b) Average Optical Densities (OD) from each band of three independent blots, measured with Image J software; values were normalized with the OD of the β-actin protein, detected using a specific antibody. Open bars: Cathepsin Z, black bars: Cathepsin B.

To relate the signal to cathepsin activity, we quantified the amounts of active cysteine proteases of the cathepsin family in the polyps as compared to healthy surrounding tissue, and control healthy intestines. To quantify specific protease activity, we used DCG-04, a biotinylated derivative of the non-specific cathepsin inhibitor JPM-565 that interacts with the active site of cysteine cathepsins [21], [22]. Tissue extracts from micro-dissected polyps and from healthy intestine tissues were incubated with DCG-04, and individual cysteine cathepsins were then identified by their relative molecular weights, after separating the extracted proteins by SDS-PAGE. This inhibitor has been used previously to measure active cathepsins B, S, L and Z (also known as cathepsin X) in cell extracts [21], [22]. We used extracts from Cathepsin B deficient mice [23], [24] as control. Comparing values from 6 mice per group confirmed significant (*P*<0.0001) up-regulation of active cathepsin B in polyps (17590±883 OD units) compared with neighboring tissue (7798±993 OD units), or from intestines of healthy age-matched mice (6879±651 OD units)(one tailed t test with Welch correction) (Fig. 5a). Treatement with anti-TNFα caused a significant drop in the levels of polyp specific active cathepsin B (7047±194 OD units; *P*<0.0001).

Similarly, we detected elevated levels of cathepsin Z in polyps (9886±971 OD) as compared with adjacent tissue (3362±752 OD units; *P* = 0.0005) or with *wt* intestine tissue (2265±595 OD units; *P* = 0.0005) (one tailed t test with Welch correction). In accordance with previous reports [25], levels of active cathepsin Z were elevated in in the intestine of Ctsb^−/−^ APC^Δ468^ mice (6364±629 OD units) as compared to *wt* intestine (2266±595 OD units) (*P* = 0.0179,t test with Welch correction). We were unable to detect cathepsin L and S. Cathepsin L has been reported to be unstable in extract [26].

### Live Imaging of Cathepsin B activity Reveals Dynamics of Polyp Growth/Regression

These observations led us to conclude that the ProSense-680 signal was reporting cancer-associated inflammation. We had previously reported that treatment of APC^Δ468^ mice with anti-TNFα results in suppression of established polyps [27]. To assess the biological impact of cathepsin-B we compared genetic ablation of cathepsin B [23], [24]. with treatment of mice with anti-TNFα. Towards this end, we crossed APC^Δ468^ mice to cathepsin_B deficient mice. Western blot analysis with DCG-04 confirmed that polyps arising in cathepsin-B ablated mice were specifically devoid of cathepsin B activity (Fig. 5a&b), while cathepsin Z was not significantly altered. Interestingly, polyps of anti-TNFα treated mice also showed a selective decrease in cathepsin B, as compared to Z (Fig 5a&b).

**Figure 6:**
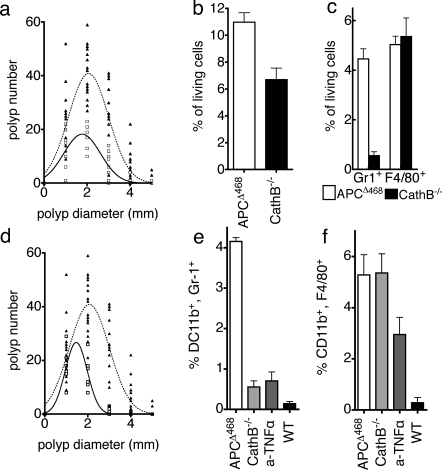
Cathepsin B deficiency or anti-TNFα treatment attenuate polyposis. (a) Non-linear regression analysis of polyp number and diameter, assuming Gaussian distribution; APC^Δ468^ Ctsb^−/−^ (continued line, open squares), and APC^Δ468^ (dotted line, closed triangles). Note that Cathepsin B^−/−^ mice had fewer and smaller polyps. (b) Frequencies of ProSense-680 active leukocytes amongst total MNCs prepared from the intestine of APC^Δ468^ (open bar, 6.7%±086%) or APC^Δ468^Ctsb^−/−^ (filled bar, 11%±0.69%, P = 0.0037; unpaired t test with Welsh correction). (c) Frequencies of ProSense-680 active CD11b^+^Gr1^+^ (mean 0.56%) or CD11b^+^F4/80^+^ (mean 4.46%) cells, from the intestines of APC^Δ468^ (open bar) and APC^Δ468^Ctsb^−/−^ mice (filled bars); P<0.001, n = 6, 2way ANOVA. Note that Cathepsin B deficiency predominantly impacted the abundance of CD11b^+^Gr1^+^ cells. (d) Attenuation of polyposis in anti-TNFα treated mice (solid line, open squares, n = 6), as compared to the APC^Δ468^ (dotted line & closed triangles). (e) Frequencies of CD11b^+^Gr1^+^ amongst total intestine live MNCs; APC^Δ468^Ctsb^−/−^ intestine (light gray bar, 0.56±0.15%, P<0.001), anti-TNFα treated (dark gray bar, 0.71±0.22%, P<0.001), untreated APC^Δ468^ (open bar, 4.2±0.093%), *wt* control intestine (black bar, 0.15±0.051%). (f) Frequencies of CD11b^+^F4/80^+^ in the APC^Δ468^ (5.03±0.78%), APC^Δ468^Ctsb^−/−^ intestine (5.36±0.92%), and anti-TNFα treated intestine (dark gray bar, 3.0±0.67%).

Both ablation of cathepsin B and treatment of mice with anti-TNFα significantly reduced polyp density and size. Cathepsin B deficient mice had less polyps (39±2.1 adenoma of Ctsb^−/−^ APC^Δ468^ mice n = 8, compared to 94±3.5 adenoma for APC^Δ468^ n = 16, *P*<0.0001 unpaired t test with Welsh correction), which had the trend to have smaller size (1.82±0.11 mm diameter for Ctsb^−/−^ APC^Δ468^ mice n = 8, compared to 2.047±0.11 mm diameter for APC^Δ468^ n = 16, *P* = 0.086) (Fig. 6a). Anti-TNFα treated mice also showed reduced numbers (34.3±4.49 adenoma, n = 8, *P*<0.0001, unpaired t test with Welsh correction) and sizes of polyps (1.516±0.022 mm in diameter, *P*<0.0001 unpaired t test with Welsh correction, n = 6) (Fig. 6c) as compared to untreated APC^Δ468^ mice.

FACS analysis revealed that intestines of Ctsb^−/−^ APC^Δ468^ mice harbored 40% less ProSense-680^+^ MNCs (6.7%±086%), as compared to the APC^Δ468^ (11%±0.69%, *P* = 0.0037, Fig. 6b). This reduction can be attributed to a drop in the levels of MDSCs with ProSense-680 activity (mean frequency 0.56% as compared to 4.46%; *P* = 0.0085, n = 6, unpaired t test with Welsh correction, Fig. 5c). The frequencies of ProSense-680^+^, CD11b^+^F4/80^+^ cells remain unchanged (mean 5.36% to 5.03% respectively). MDSC density was also markedly reduced in anti-TNFα treated mice (Fig 6e), although with this treatment macrophages also showed a significant drop in frequency (Fig 6f).

**Figure 7:**
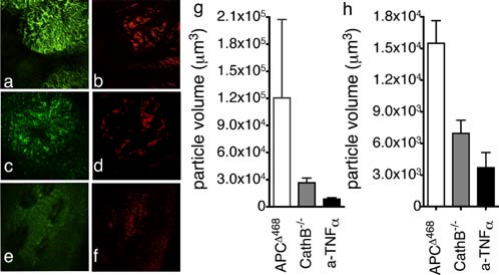
Imaging polyps in mice deficient for Cathepsin B or responding to effective anti-TNFα therapy. Spectral separation of images from, (a&b) APC^Δ468^ mice, (c&d) Ctsb^−/−^APC^Δ468^, (e&f) anti-TNFα treated APC^Δ468^ mice; ProSense-680 (green; CathepsinB), AngioSense-750 (red; blood vessels). The objective used was the UplanApo 4× (pixel size 5.4 µm, lateral resolution 12.42 µm). (g) Mean particle volumes in the Z stacks of fluorochrome 680 (open bar: APC^Δ468^, 120554±86906 µm; gray bar: APC^Δ468^Ctsb^−/−^, 26609±5268 µm^3^; anti-TNFα treated APC^Δ468^, 8639±1570 µm^3^; P<0.0001 one sample test). (h) Mean particle volumes in the Z stacks of fluorochrome 750 (open bar: APC^Δ468^, 15501±2144 µm^3^; gray bar: APC^Δ468^Ctsb^−/−^, 6963±1236 µm^3^, anti-TNFα treated, 3700±1444 µm^3^; P<0.0001 one sample test).

To relate changes in ProSense-680^+^ MNCs in treated mice with changes in *in situ* imaged fluorescent signal, we imaged polyps from these mice and analysed the z-stacks in a quantitative manner. The volumes of cathepsin ProSense-680^+^ particles were significantly smaller in Ctsb^−/−^ APC^Δ468^ polyps (mean volume 26609±5268 µm^3^, *P*<0.0001, one sample t test, Fig. 7 g&c) and polyps in anti-TNFα treated APC^Δ468^ mice (mean volume 8639±1570 µm^3^, *P*<0.0001, one sample t test, Fig. 7 g&e) as compared with polyps in age matched APC^Δ468^ mice (mean volume 120554±86906 µm^3^, Fig. 7 g&a). The numerical results are in accordance with the optical impression of the images, and with the intesity of signal being higher in APC^Δ468^ than Ctsb^−/−^ APC^Δ4688^ and the anti-TNFα treated APC^Δ468^ (Fig 7).

The mean volume of the vessels stained with AngioSense 750 was 2.2 times smaller in Ctsb^−/−^ APC^Δ468^ adenoma (6963±1236 µm^3^, Fig. 7 d&h), and 4.2 times smaller in the anti-TNFα treated APC^Δ468^ adenoma (3700±1444 µm^3^, Fig. 7 f&h), as compared to the polyps in APC^Δ468^ mice (15501±2144 µm^3^, Fig. 7 b&h).

These observations indicate that near infra red activity-based ProSense-680 accurately marks areas of dysplasia, preferentially reports cathepsin B activity, and reveals biological activity directly linked with the progression or regression of the polyps. Furthermore, the AngioSense 750 signal provided us with independent confirmation of tumor associated angiogenesis, the location of dysplasia, and biological response to treatment.

## Discussion

Endoscopic techniques have provided sensitive means of detecting adenomatous polyps that could potentially progress to colon cancer. Thus, endoscopic monitoring of susceptible individuals has made it possible to detect and remove early dysplastic lesions before they become life threatening. To date, visualization of the dysplasia has been limited to detection of anatomical abnormalities, and there is no precedence of live imaging of biological activity that is predictive of tumor growth versus regression. In this study we have shown that cathepsin B activity is an inherent component of cancer-associated inflammation in tumor infiltrating myeloid cells. We have demonstrated that live imaging of this activity is feasible, and provides for accurate demarcation of dysplasia and the associated neo-angiogenesis. Furthermore, we have demonstrated that this mode of imaging reveals dynamics of biological activity that is predictive of tumor progression versus response to therapy.

Cathepsin B activity has been associated with a number of tumors in humans and experimental animals, making it an attractive avenue for the imaging and possible treatment of cancer [10], [19], [20]. Here we provide evidence that in addition to cathepsin B, another member of the cysteine family of cathepsin Z is significantly up regulated in adenomatous polyps. Cathepsin activities were focal and readily distinguished the dysplastic lesions from the healthy neighboring tissue. Thus, imaging of this activity using protease sensitive probes allows accurate detection of areas of dysplasia that may be undetectable by visible light imaging, due to size limits or anatomical features.

The source of cathepsin activity in tumors has been in the past debated, with some reports emphasizing that tumor cells are the major source of this activity. Here, we provide evidence based on histology and flow cytometry that clearly reveals tumor infiltrating myeloid lineage cells as the predominant source of this activity. This is a fortunate and useful finding, as cancer-associated inflammation is causatively linked with adenoma growth. Significantly, our analysis of fluorescent signals detected with in the lesions revealed that the local increase in signal intensity was due to increased numbers of cathepsin active cells rather than higher activity per cell. Furthermore, we could confidently and accurately detect this activity to a depth of at least 75 µm. Thus, the imaged signal was directly reporting the abundance of pro-inflammatory cells within the dysplasia.

We demonstrated that genetic ablation of cathepsin B results in suppression of tumor infiltrating pro-inflammatory cells, notable attenuation of polyposis, and a decrease in the fluorescent signal emanating from the lesions. Ablation of cathepsin B significantly increased the overall levels of active cathepsin Z, Previous reports suggest that cathepsin Z (also called cathepsin X) compenstates for the levels of membrane bound cathesin B, and is elevated in cathpesin B knock out mice [25]. Thus, attenuation of probe signal in polyps arising in cathepsin B knock mice suggests that the probe activity was relatively specific for cathepsin B. Furthermore, attenuation of polyposis in these mice suggests a specific requirement for cathepsin B in the progressive growth of polyps.

Surprisingly, cathepsin B deficiency predominantly affected the CD11b^+^Gr1^+^ MDSC infiltrate, and did not impact the CD11b^+^F480^+^ macrophage component of polyp infiltrating leukocytes. Both of these myeloid cell types contributed equally to the local activation of the ProSense 680 probe. Since preferential loss of MDSC correlated with polyp attenuation, we conclude that these cells were critically contributing to the progressive growth of dysplasia. Furthermore, we conclude that the decrease in cathepsin activated probe signal was largely due to the loss of MDSCs from areas of dysplasia.

The cytokine TNFα is regarded to be at the apex of inflammatory responses, promoting angiogenesis, mobilization of neutrophils and escalation of inflammation [28], [29], including cancer associated inflammatory reactions [30]. Treatment of mice with anti-TNFα suppresses pathogen induced inflammatory bowel disease and inflammation triggered cancer [27], [31], [32]. We therefore postulated that if pro-inflammatory cells were the source of cathepsin activity and played a causative role in polyposis, then suppression of polyposis-associated inflammation should hinder progressive polyp growth, and this therapeutic effect should be reflected in a significant down-regulation of cathepsin activity. Treatment of mice with anti-TNFα resulted in a preferential loss of MDSC infiltrating the lesion, in increased apoptosis of the aberrant epithelial cells, and regression of the lesions. Accordingly, cathepsin-B activities as measured by western blot analysis and probe signal were significantly attenuated. Altogether, these observations establish live imaging of cathepsin B activity with sensitive near infrared probes as a highly specific method for detection of biological activity linked with progressive tumor growth.

Tumor associated neo-angiogenesis is considered to be a necessary pathological component of tumor growth and a viable target for therapeutic intervention. Pro-inflammatory cells are a recognized source of angiogenic factors, and suppression of inflammation is therefore expected to impact tumor associated inflammation. It is not known at what stage dysplasia triggers angiogenesis and how the dynamics of infiltrating leukocytes reflect the expansion or regression of blood vessels in the lesion. Here, taking advantage of a constitutively active near infrared probe we have imaged in a living animal the neo-vascularization of early dysplastic lesions by micro-vessels, and revealed the change in architecture of the vasculature entering the lesion. Using different fluorescent excitation and spectral separation of the images we succeeded in simultaneously imaging both angiogenesis and cathepsin B activity in the same tissue and 3-dimensional space. The visualization of the architecture of the lesion was further enhanced by concomitant imaging of auto-fluorescent signals that emanated largely from the tumor mucosa

We were able to demonstrate that anti-inflammatory regiments such as ablation of cathepsin B or treatment of mice with anti-TNFα impacts the infiltration of dysplastic lesions by the microvasculature, and that loss of the vessels correlates with attenuation or regression of the lesions. Thus, near infrared imaging of blood microvasculature provides an independent means of monitoring progressive dysplasia, and response to effective therapeutic intervention.

In summary, we have shown that live imaging of mechanism-based near infrared probes allow simultaneous detection of independent biological activities that report progressive tumor growth. Our earlier studies have documented the feasibility of application of this mode of imaging to endoscopic monitoring of cancerous lesions. Here, we have provided mechanistic information on the source and biological significance of the signal. All together, we have documented a powerful advance in our visualization of tumor biology dynamics, which allows for sensitive monitoring cancer progression or favorable response to therapy.

## Materials and Methods

### Microscope and the operational organization

The prototype Olympus IV 100 LASER scanning intravital microscope was used to collect images from living tissues. This microscope has 4 lasers that with the use of a specialized array of filters can excite a wide variety of fluorochrome including the Cy5.5 (max excitation 680 nm, excited in this microscope with the 633 nm LASER, emission 680 nm) and Cy7 (excitation 749 nm, emission 790 nm). For this study the 4× and the 10× dry objectives were used. The emissions were separated in the desired spectrum through a set of mirrors and filters and collected from 3 Photomultipliers (PMT). Olympus FlowView software is used to compensate the channels and collect the FlowView images.

The microscope uses motors controlled by the software for the collection of the z-stacks used in this report. A 37°C table was available for the comfort of the anesthetized animals during operation.

### Live imaging probes

To visualize in detail the cathepsin activity and its distribution along the intestine three probes were utilized, the ProSense 680 (ProSense 680), ProSense control 750 and a high molecular weight (250,000 g/molecule) probe linked with fluorochrome that is excited at 750 nm and emits at 790 nm (AngioSense750). ProSense 680 is a composite probe that is based on a poly-lysine chain on which a number of fluorochrome molecules were linked on the side chain of the lysine moiety, as well as a number of MPEG molecules. The composite probe with the poly-L-lysine backbone intact does not emit fluorescence because of quenching, but when the backbone is hydrolyzed by cathepsins the fluorochrome emits a strong signal. ProSense control 750 has a poly-D-lysine backbone that cannot be hydrolysed by cathepsins and therefore remains quenched and it is used as probe to visualize the non specific quenched accumulation of fluorescent dyes in areas of high vascularity. AngioSense 750 remains in the vessels during the normal experimentation period and delineates the vessels. The autofluorescent signal (505–510 nm) was used to visualize the outline of the tissue imaged.

### Mouse studies

Cathespin B knock-out mice have been reported before and were a kind gift [23], [24]. Throughout the procedures in which animals were injected with the probe, as well as during the imaging session, mice were anaesthetized with inhalation of 1.5–2% mixture of Isoflurane in oxygen.

Mice were retro-orbitally injected with the ProSense-680 24 hours before the experimental procedure (2 nm/mouse, 150 µl). Ten minutes before the imaging session the mice were injected with 100 µl of the AngioSense-750. According to our observations most of the probe remains in the vessels for at least 1 hour.

Mice were incised and a loop of the intestine was cut along the length and opened to allow imaging in z stacks from the inside out. The mice were constantly monitored for the rate of the respiration and the depth of their anesthesia. The typical imaging session has duration of 60–90 min. throughout the imaging session the mouse is alive and anaesthetized.

All animal experiments were approved by the Harvard Medical School Standing Committee on Animals (protocol 04084, Dr K. Khazaie), by the Dana Farber Cancer Institute IACUC (protocol 02033, Dr Khazaie) and the Northwestern University Animal Study Protocol 2007-1284 (Dr Khazaie) for “imaging proteolytic activity in colon cancer”.

### Imaging analysis software

The collected z-stacks were in the form of multiple “. tiff” FlowView files and can be seen and split into channels with the USDA plug-in collection of ImageJ open source software. To analyze the particles we used the bundle of plug-in developed by the Bob and John Wright Cell Imaging Facility of the University of Ontario Canada (http://www.uhnresearch.ca/facilities/wcif/fdownload.html). Several other stack plug-ins were utilized.

### Statistical analysis

Any values referred to as statistically analyzed are expressed as “mean value±SEM”. We consider a *P* value between groups to be significant if *P*<0.05.

For the preparation of Fig. 3i we employed Linear Regression Analysis to correlate the size of the particles with their total intensity. We used unpaired t test with Welsh's correction analysis to correlate the sets of values of intensity per voxel in the particles of the APC^Δ468^ adenoma and the wt images, for the comparison of the frequencies of the ProSense-680^+^ stained leukocytes (Fig. 6b) and the correlation of the frequencies of the MDSCs ProSense-680^+^ cells in APC^Δ468^ and *wt* MNCs. We employed one sample t test to compare the size of the ProSense-680^+^ particles of APC^Δ468^ adenoma with the ProSense-680^+^ particles in Ctsb^−/−^ APC^Δ468^ and TNFα treated APC^Δ468^ polyps because the size of particles was very diverse in all samples (Fig. 7).

For the preparation of Fig. 6a&c we employed Non-Linear Regression Analysis to correlate the size of the polyps with their number assuming that the size distribution is Gaussian. The non-linear regression graph was superimposed with the actual values of the size and the numbers of the polyps in each individual mouse. “Prism 4” software was used for all these analyses, and the related graphs.

### Immunofluorescence analysis

Mice were stained in vivo for 24 hours with 2 nm ProSense-680. The following day were sacrificed and the fillet-opened intestine was rolled and frozen in OCT. Cryosections were prepared (15 µm) and fixed in acetone (−20°C, 15 min), rehydrated in PBS and incubated with the primary antibodies (biotinylated anti-CD11b, 1∶75, and purified rat anti-Gr1, 1∶75) antibodies for one hour. The slides were washed in PBS (3×5 min each) and incubated with the secondary antibodies (streptavidin AlexaFluor 594, and anti-rat IgG AlexaFluor 488, 1∶75 each). After an hour the sections were washed with PBS and mounted with antifade mounting solution that contains DAPI. A specific filter was utilized to visualize the Cy5.5 fluorochrome

## Supporting Information

Figure S1 *Distribution of adenomas in the APC^Δ^* ^468^ *intestine*. (a) the number of polyps plotted against their location throughout the length of the small intestine of 3 month-old mouse. (b) Typical distribution of polyps along the length of the small intestine. (c) Number of polyps as a function of age of mice, plotted in three size groups. (d) Number of polyps as a function of age of mice.
(3.76 MB TIF)

Figure S2 *Histological properties of APC^Δ468^ polyps*. (a) β-galactosidase staining APC^Δ468^ adenoma. (b) PAS staining of a 6 µm paraffin section of an APC^Δ468^ adenoma. The arrow indicates the characteristic purple staining of the mucus inside the goblet cells. (c) X-gal staining of TS4cre APC^lox468^ R36R polyp; (d) H&E staining of a jellyroll preparation of an APC^Δ468^ intestine. Magnification 50×. The arrow indicates a large adenoma. (e) 100× magnification of an area of the same section. The arrow indicates a small adenoma and the arrowhead a normal villus. (f) 400× magnification of the area surrounded with the white rectangular in (e). The arrow indicates an adenoma.
(3.95 MB TIF)

Figure S3 *Systemic inflammation in the APC^Δ^* ^468^ *mice*. (a) Increase in the weight of the spleen as the mice age (blue dots) as compared to the wt spleen weight (red dots) (P = 0.0003). The CD11b^+^Gr1^+^ (b) and the CD11b^+^F4/80^+^ cell numbers in the spleen of the APC^Δ468^(open bars) and the wt (black bars) mice.
(0.47 MB TIF)

Figure S4 *Autofluorescence intensity of the APC^Δ468^ adenoma*. The distribution of the auto fluorescence alongside the z-stack. APC^Δ468^ adenoma shown in Fig. 3f (black squares) as compared to the wt equivalent shown in Fig. 3e (open triangles).
(0.30 MB TIF)

Figure S5 *Cathepsin activity is required for unquenched ProSense 680 signal in the APC^Δ468^ adenoma*. APC^Δ468^ mice were stained a day before the imaging session with 2 nmoles/mouse of ProSense 680 and ProSense control 750. Stacks of 168 (1 µm/slice) slices were collected using the Olympus IV100, the UplanApo ×4 and ×2.5 electronic zoom using the channels for ProSense 680 and ProSense control 750. a. merge of the two channels of the160th slice; b. the ProSense 680 image of the160th slice; c. the ProSense control 750; d. the ratio of the ProSense 680 to the ProSense 750 signal. The mean intensity of the adenoma Region Of Interest (ROI, left rectangular) and the healthy surrounding ROI (right rectangular) was measured for the both channels in all 168 slices, The bar diagram shows that the this ratio is 49±1.4 for the adenoma ROI and 2.1±0.01 for the healthy. The sets of values are statistically significant (*P*<0.0001, t test with Welch correction).
(1.06 MB TIF)

Figure S6 *The use of the JPM-565 inhibits the ex vivo staining of the MNCs with ProSense 680*. Isolated APC^Δ468^ MNCs were pretreated with 50 µg/ml of JPM-565 in RPMI 1640 for an hour in 37°C and 5% CO_2_. The positive control remained in the incubator treated with the carrier of the JPM-565. The cell suspension was then stained with 0.2 nmoles ProSense 680. The stained cells were stained with PE anti CD11b antibody and DAPI to detect the dead cells. a. represents the flow cytometry of the ProSence 680^+^ CD11b^+^ cells untreated with JPM-565 and b. the treated equivalent.
(0.45 MB TIF)

Video S1 *Video of the z-stack of an APC^Δ468^ adenoma*. The stack consists of 72 RGB images obtained in three channels. Autofluorescence at 505–510 nm (blue), ProSense-680 at 694 nm (green), and vascularity contrast enhancing AngioSense-750 at 790 nm (red).
(4.47 MB MOV)
