# Correction: Live Imaging of Cysteine-Cathepsin Activity Reveals Dynamics of Focal Inflammation, Angiogenesis, and Polyp Growth

**DOI:** 10.1371/annotation/499b225f-e661-4124-aa2f-60bef89bd14a

**Published:** 2008-10-23

**Authors:** Elias Gounaris, Ching H. Tung, Clifford Restaino, René Maehr, Rainer Kohler, Johanna A. Joyce, Hidde L. Ploegh, Terrence A. Barrett, Ralph Weissleder, Khashayarsha Khazaie

The seventh author's name was spelled incorrectly. It should read: Hidde L. Ploegh.

