# Supplementary figures and images for: Live Imaging of Cysteine-Cathepsin Activity Reveals Dynamics of Focal Inflammation, Angiogenesis, and Polyp Growth
Source: PLoS One. 2008 Aug 13;3(8):e2916. doi: 10.1371/journal.pone.0002916 (PMC2488397; doi:10.1371/journal.pone.0002916)

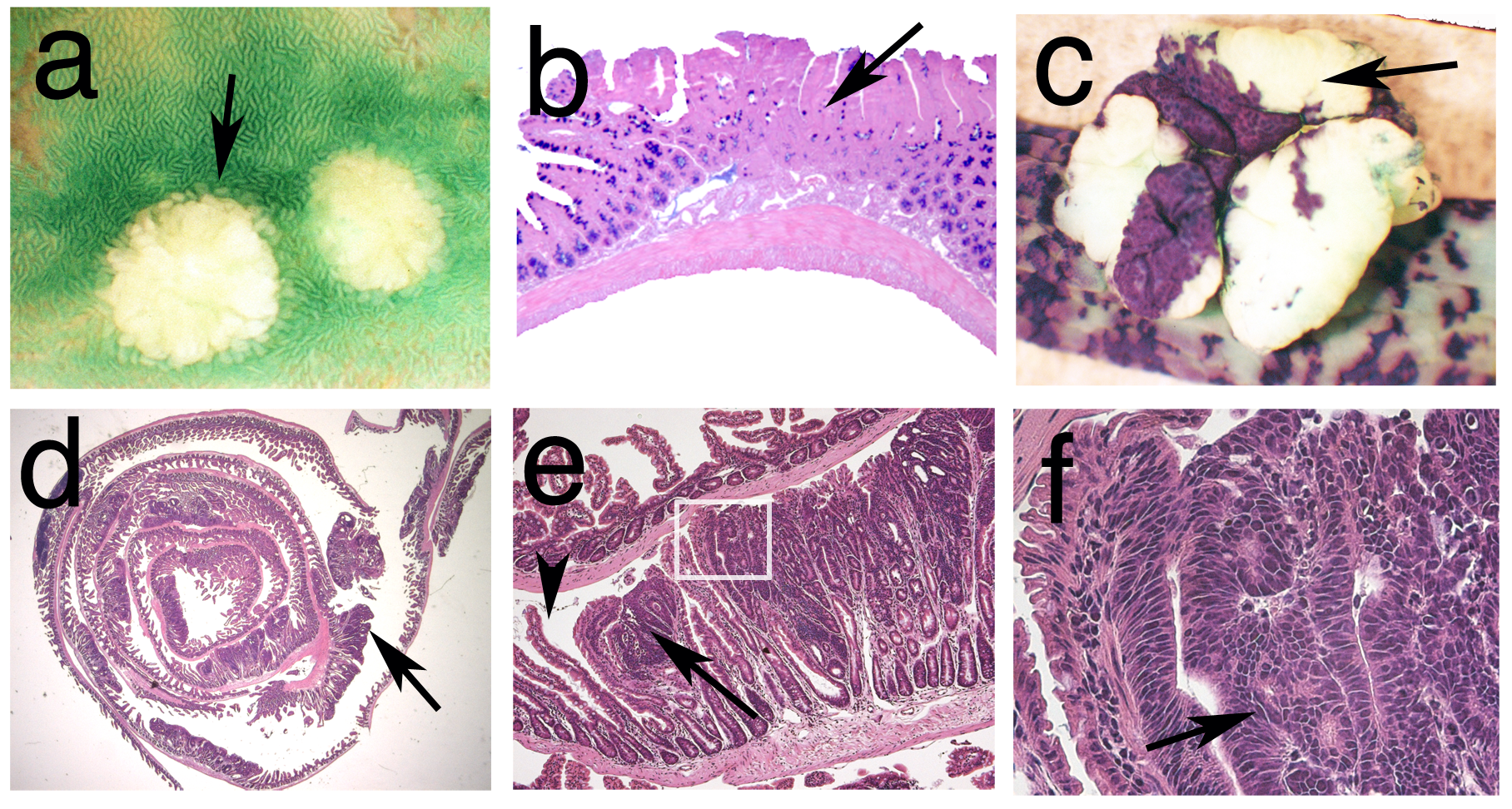

Supplement: Figure S2 — Histological properties of APCΔ468 polyps. (a) β-galactosidase staining APCΔ468 adenoma. (b) PAS staining of a 6 µm paraffin section of an APCΔ468 adenoma. The arrow indicates the characteristic purple staining of the mucus inside the goblet cells. (c) X-gal staining of TS4cre APClox468 R36R polyp; (d) H&E staining of a jellyroll preparation of an APCΔ468 intestine. Magnification 50×. The arrow indicates a large adenoma. (e) 100× magnification of an area of the same section. The arrow indicates a small adenoma and the arrowhead a normal villus. (f) 400× magnification of the area surrounded with the white rectangular in (e). The arrow indicates an adenoma. (3.95 MB TIF) [file pone.0002916.s002.tif]

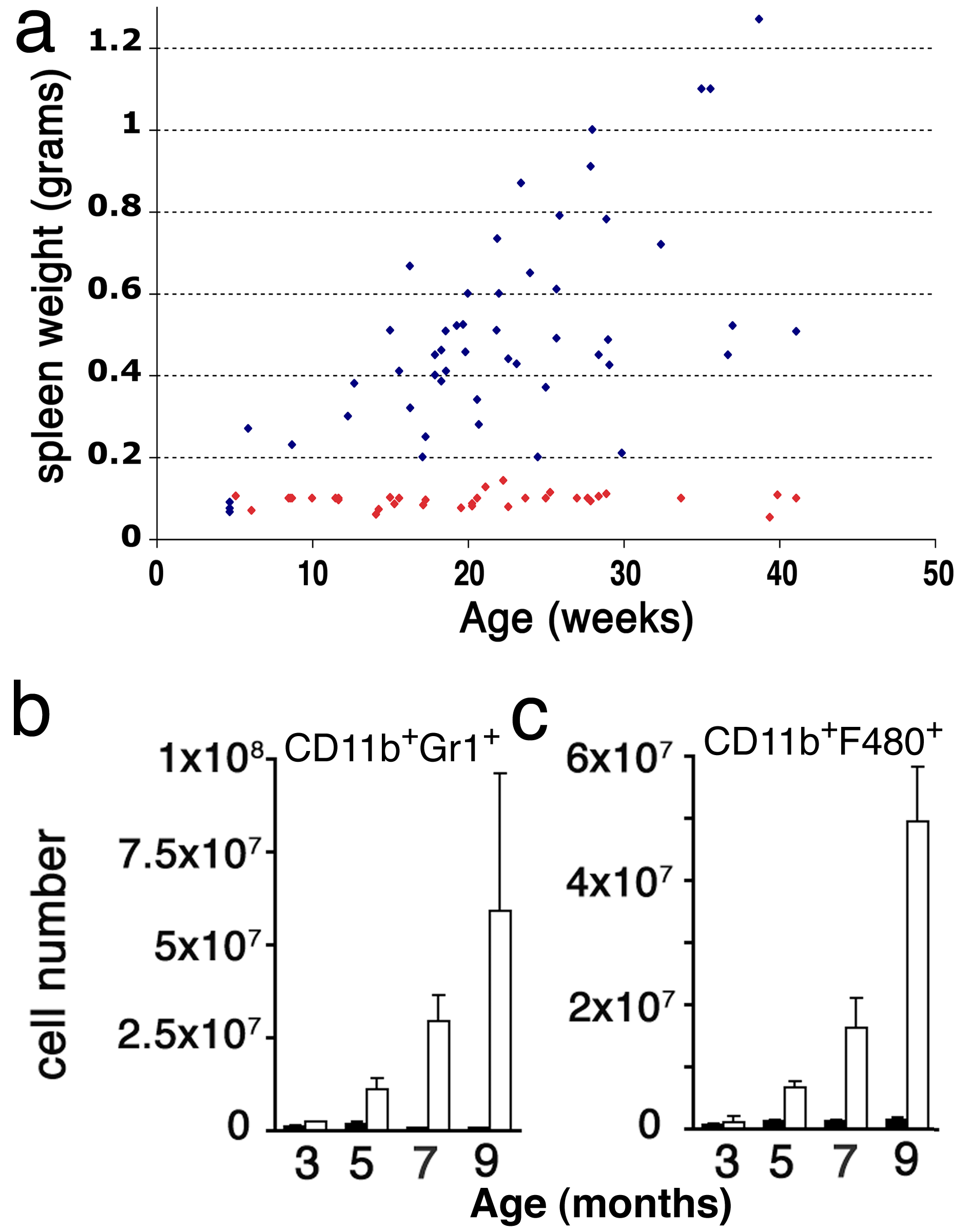

Supplement: Figure S3 — Systemic inflammation in the APCΔ 468 mice. (a) Increase in the weight of the spleen as the mice age (blue dots) as compared to the wt spleen weight (red dots) (P = 0.0003). The CD11b+Gr1+ (b) and the CD11b+F4/80+ cell numbers in the spleen of the APCΔ468(open bars) and the wt (black bars) mice. (0.47 MB TIF) [file pone.0002916.s003.tif]

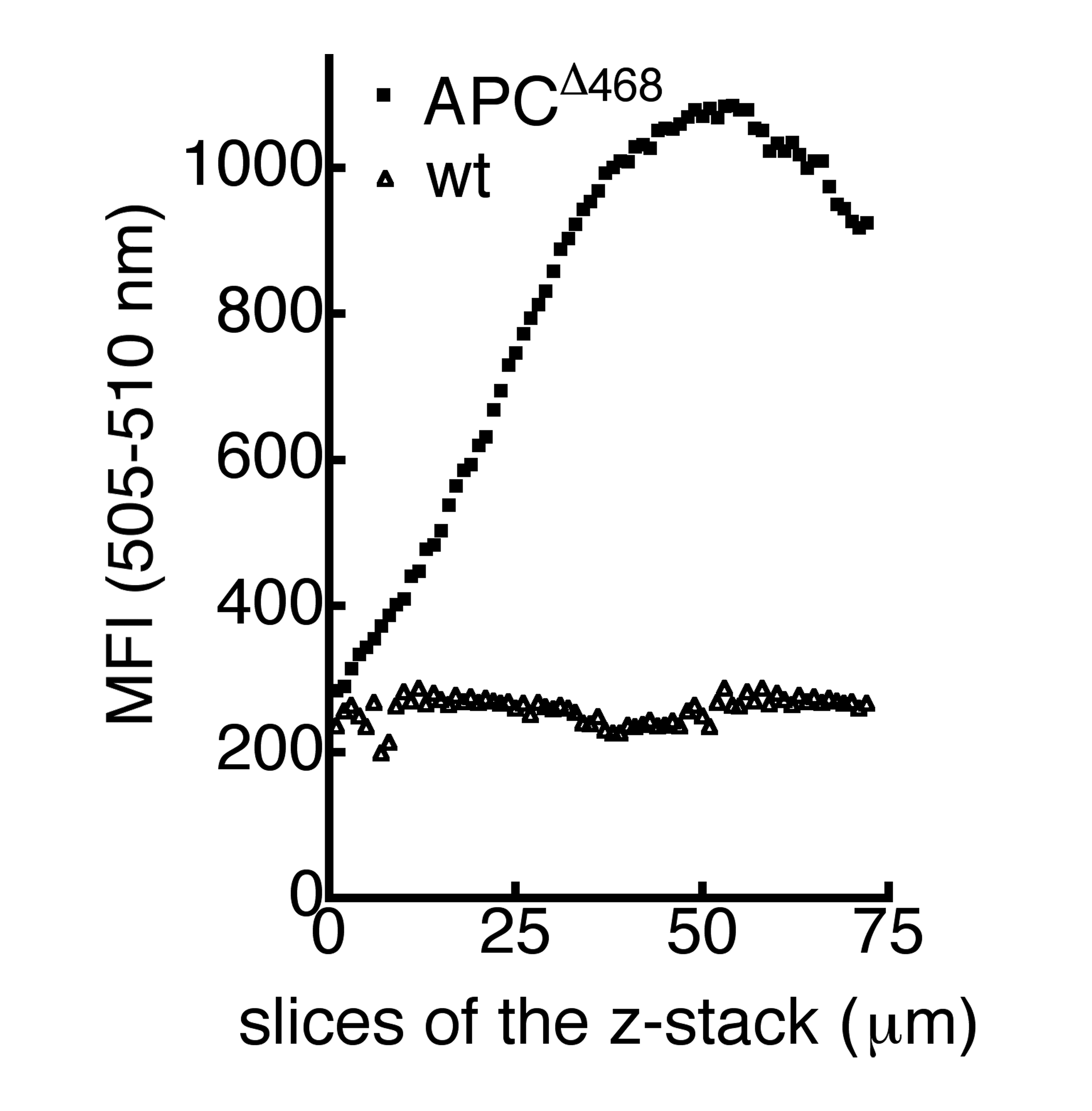

Supplement: Figure S4 — Autofluorescence intensity of the APCΔ468 adenoma. The distribution of the auto fluorescence alongside the z-stack. APCΔ468 adenoma shown in Fig. 3f (black squares) as compared to the wt equivalent shown in Fig. 3e (open triangles). (0.30 MB TIF) [file pone.0002916.s004.tif]

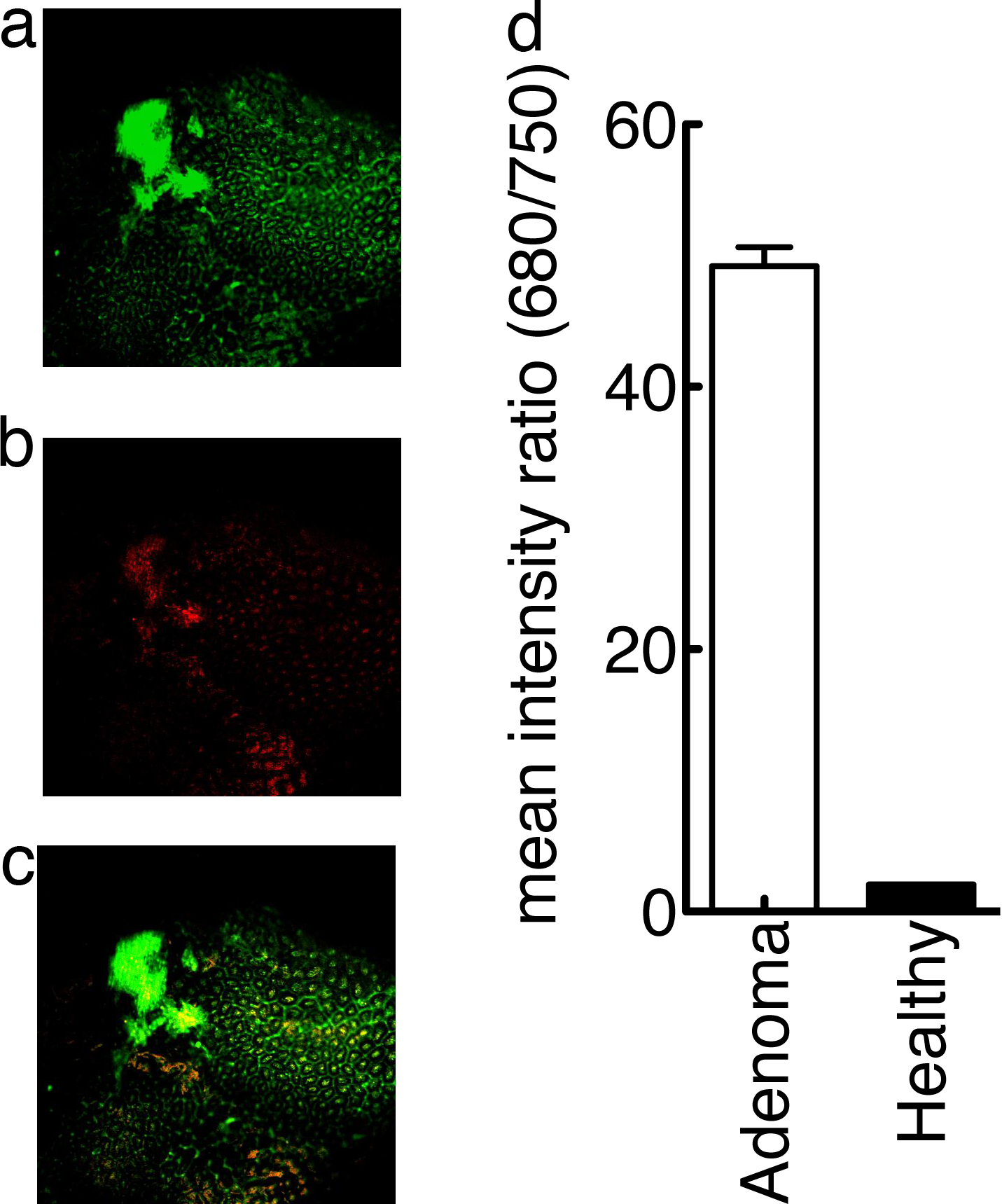

Supplement: Figure S5 — Cathepsin activity is required for unquenched ProSense 680 signal in the APCΔ468 adenoma. APCΔ468 mice were stained a day before the imaging session with 2 nmoles/mouse of ProSense 680 and ProSense control 750. Stacks of 168 (1 µm/slice) slices were collected using the Olympus IV100, the UplanApo ×4 and ×2.5 electronic zoom using the channels for ProSense 680 and ProSense control 750. a. merge of the two channels of the160th slice; b. the ProSense 680 image of the160th slice; c. the ProSense control 750; d. the ratio of the ProSense 680 to the ProSense 750 signal. The mean intensity of the adenoma Region Of Interest (ROI, left rectangular) and the healthy surrounding ROI (right rectangular) was measured for the both channels in all 168 slices, The bar diagram shows that the this ratio is 49±1.4 for the adenoma ROI and 2.1±0.01 for the healthy. The sets of values are statistically significant (P<0.0001, t test with Welch correction). (1.06 MB TIF) [file pone.0002916.s005.tif]

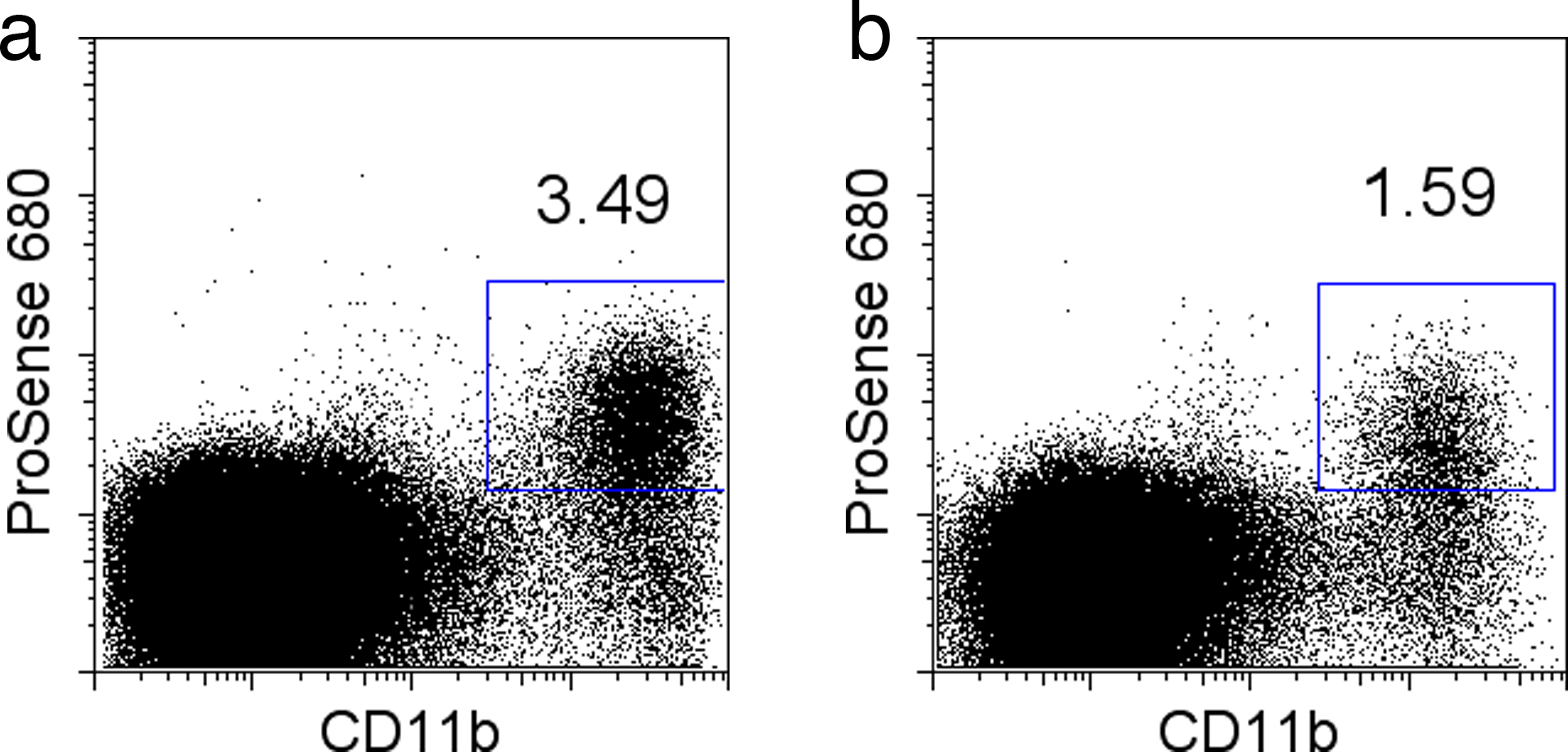

Supplement: Figure S6 — The use of the JPM-565 inhibits the ex vivo staining of the MNCs with ProSense 680. Isolated APCΔ468 MNCs were pretreated with 50 µg/ml of JPM-565 in RPMI 1640 for an hour in 37°C and 5% CO2. The positive control remained in the incubator treated with the carrier of the JPM-565. The cell suspension was then stained with 0.2 nmoles ProSense 680. The stained cells were stained with PE anti CD11b antibody and DAPI to detect the dead cells. a. represents the flow cytometry of the ProSence 680+ CD11b+ cells untreated with JPM-565 and b. the treated equivalent. (0.45 MB TIF) [file pone.0002916.s006.tif]
